# Supplementary material for: Synthesis and biological evaluation of small molecule modulators of CDK8/Cyclin C complex with phenylaminoquinoline scaffold
Source: PeerJ. 2020 Mar 13;8:e8649. doi: 10.7717/peerj.8649 (PMC7075364; doi:10.7717/peerj.8649)

^{1}H NMR spectrum of compound **5a**

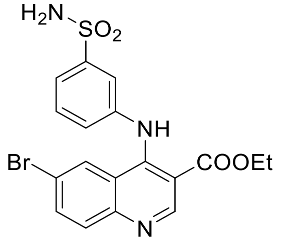

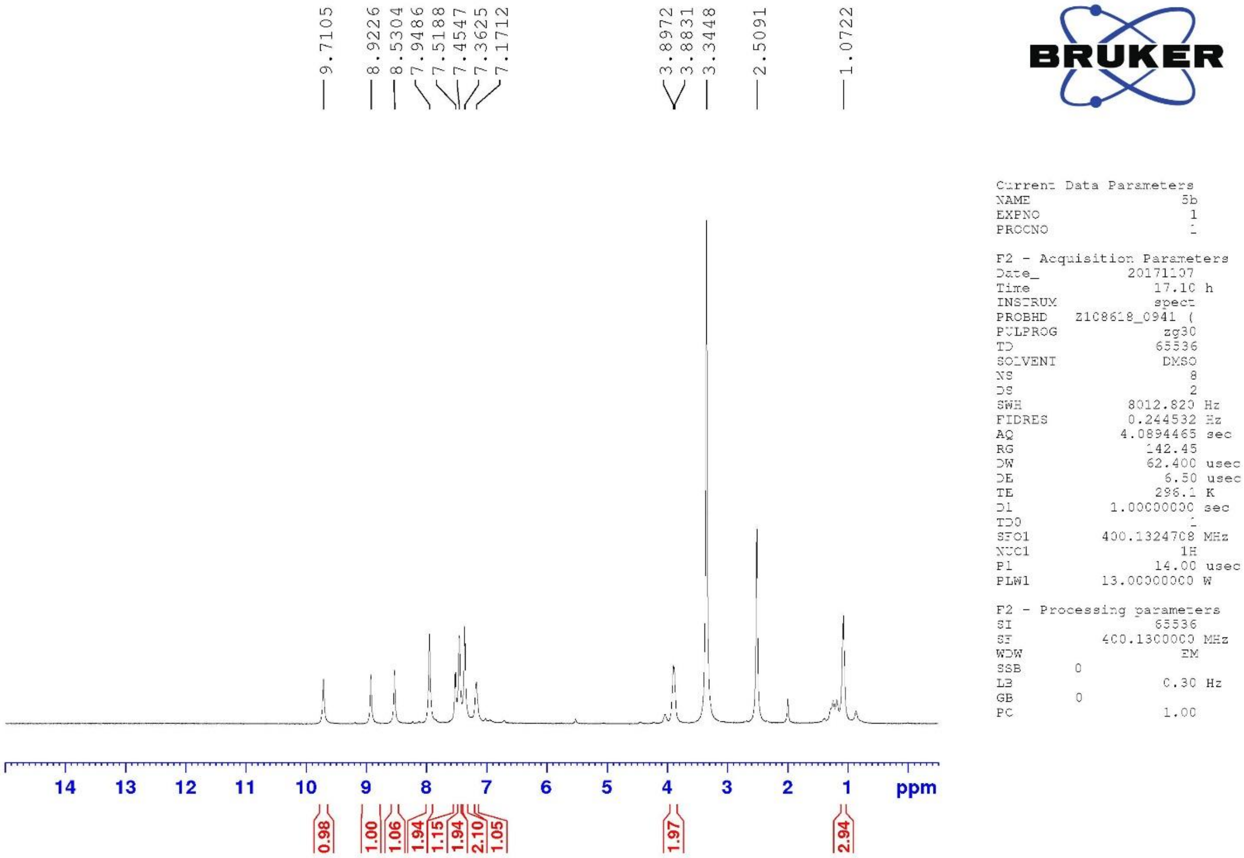

^{13}C NMR spectrum of compound **5a**

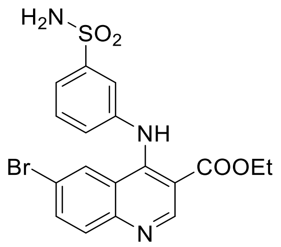

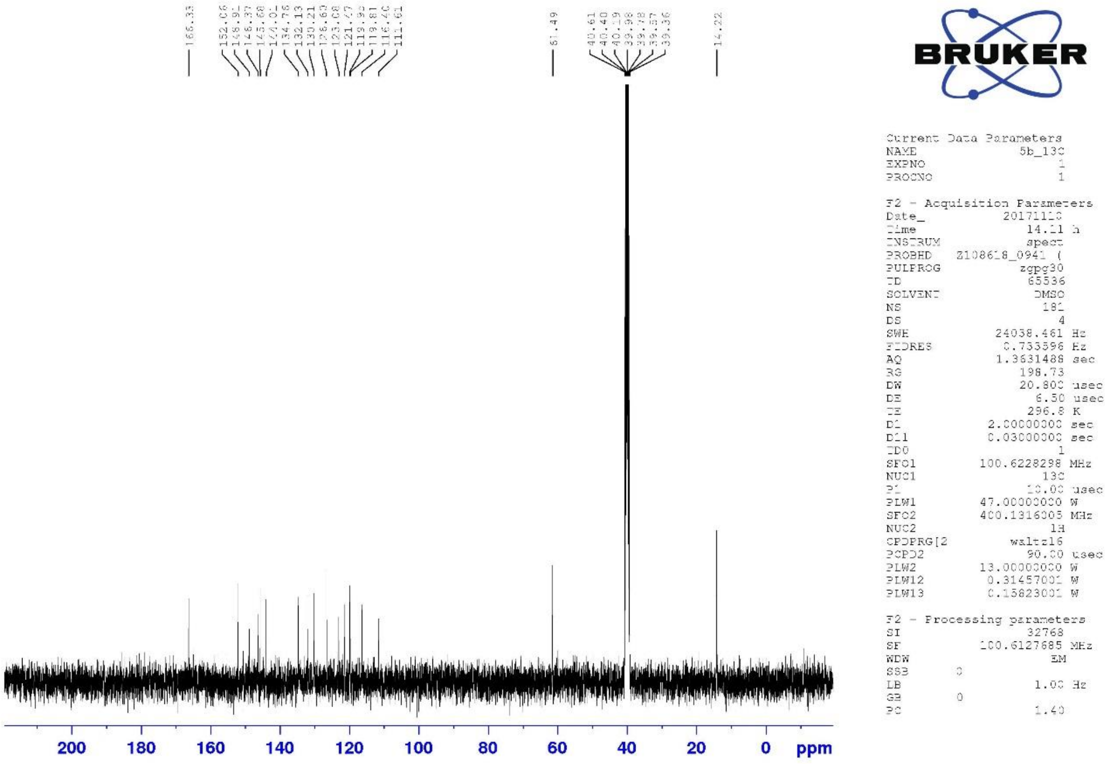

Purity and HRMS of compound **5a**

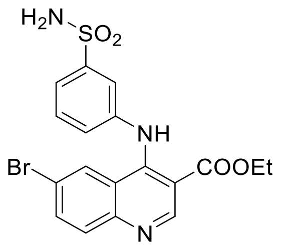

2. 5b : chromatogram / area

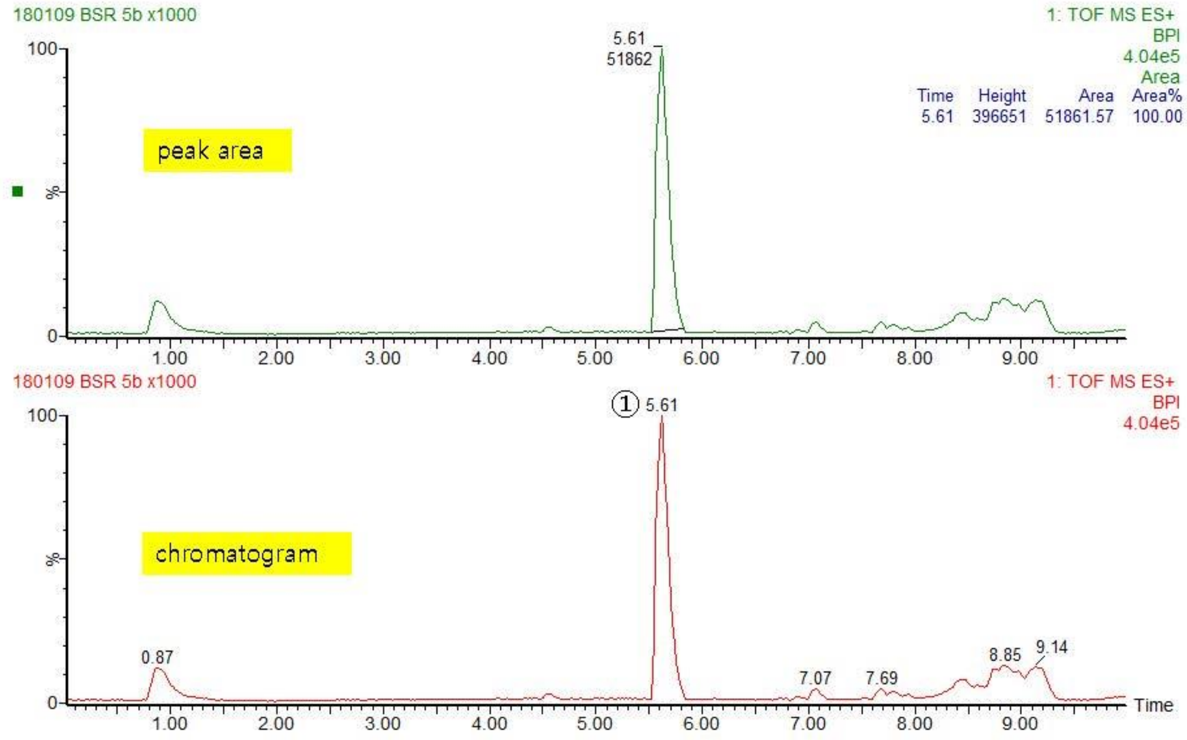

2. 5b : mass spectrum

Molecular formula: $C_{18}H_{16}BrN_3O_4S$
Exact mass: 449.0045
113.2589 (4+)
150.6760 (3+)
225.5101 (2+)
450.0123 (1+)

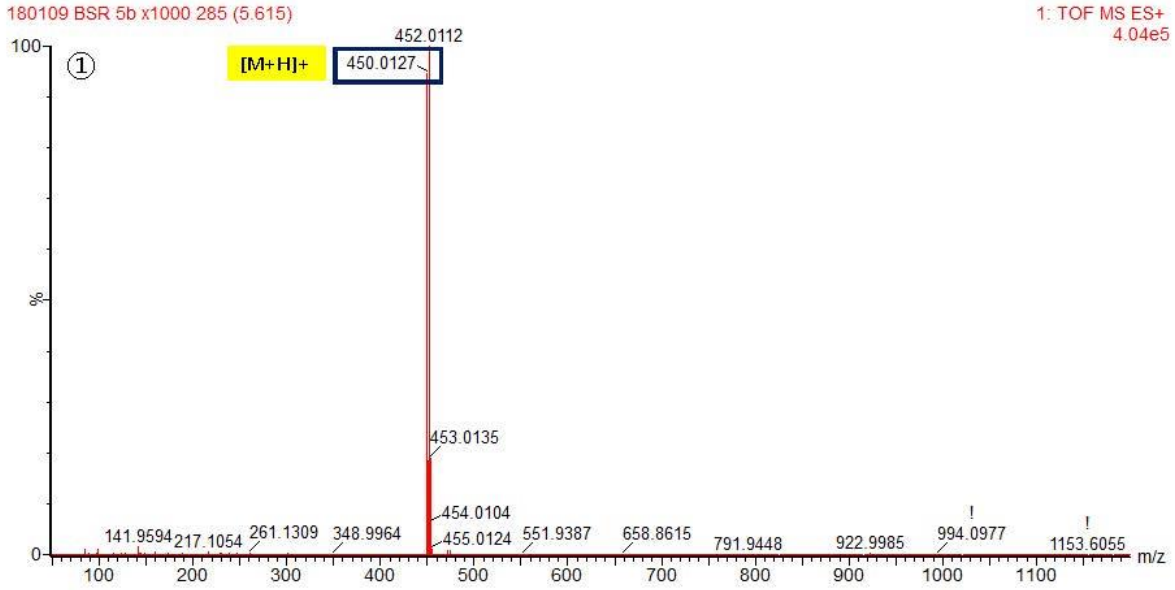

[1] NMR spectrum of compound **5b**

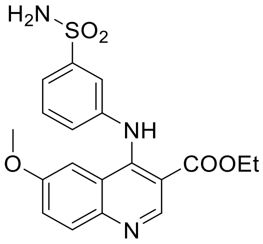

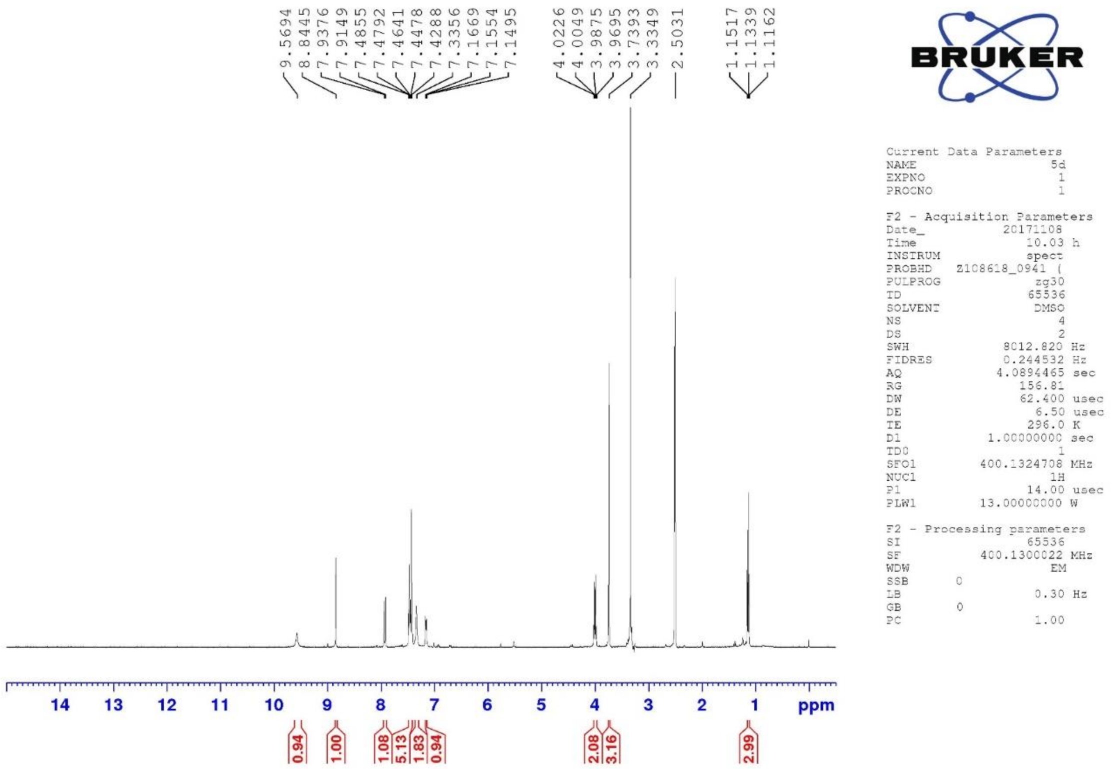

^{13}C NMR spectrum of compound **5b**

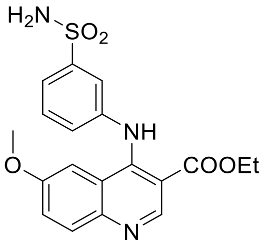

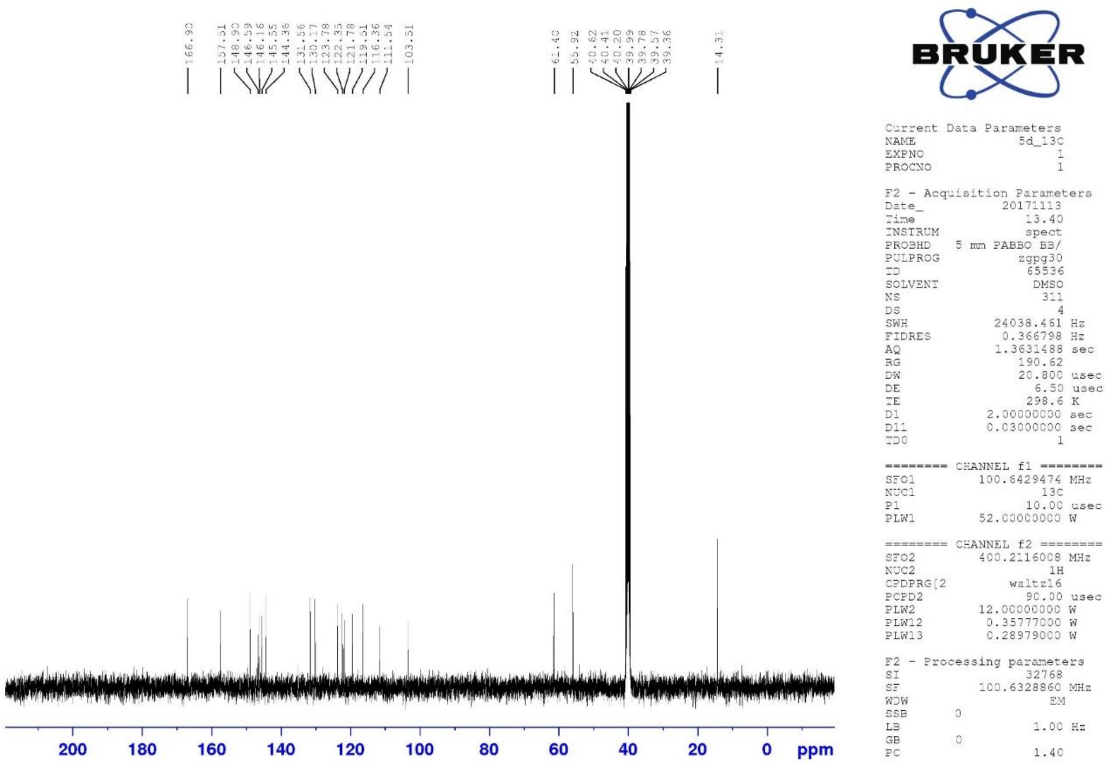

Purity and HRMS of compound **5b**

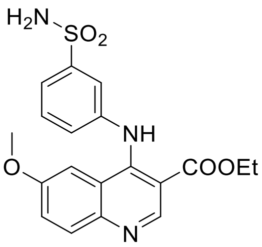

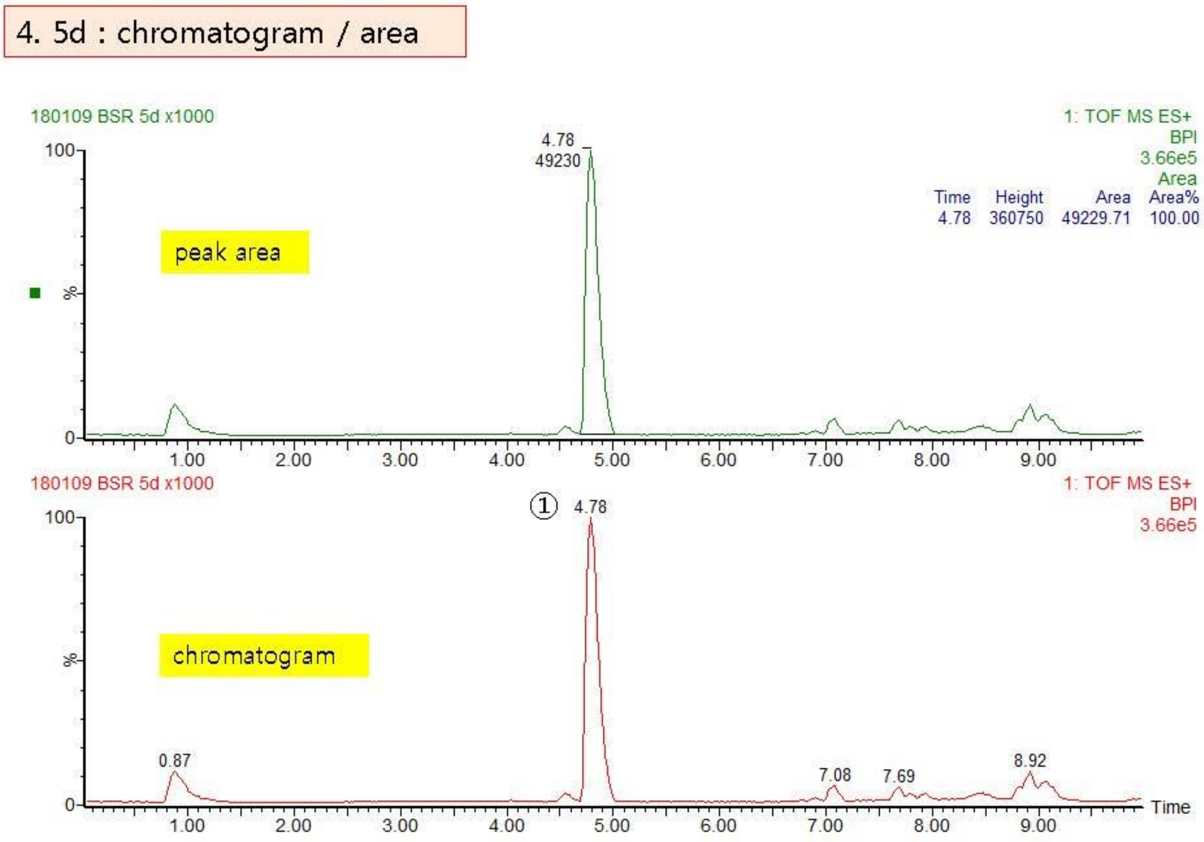

4. 5d : mass spectrum

Molecular formula: $C_{19}H_{19}N_3O_5S$
Exact mass: 401.1045
101.2840 (4+)
134.7093 (3+)
201.5601 (2+)
402.1124 (1+)

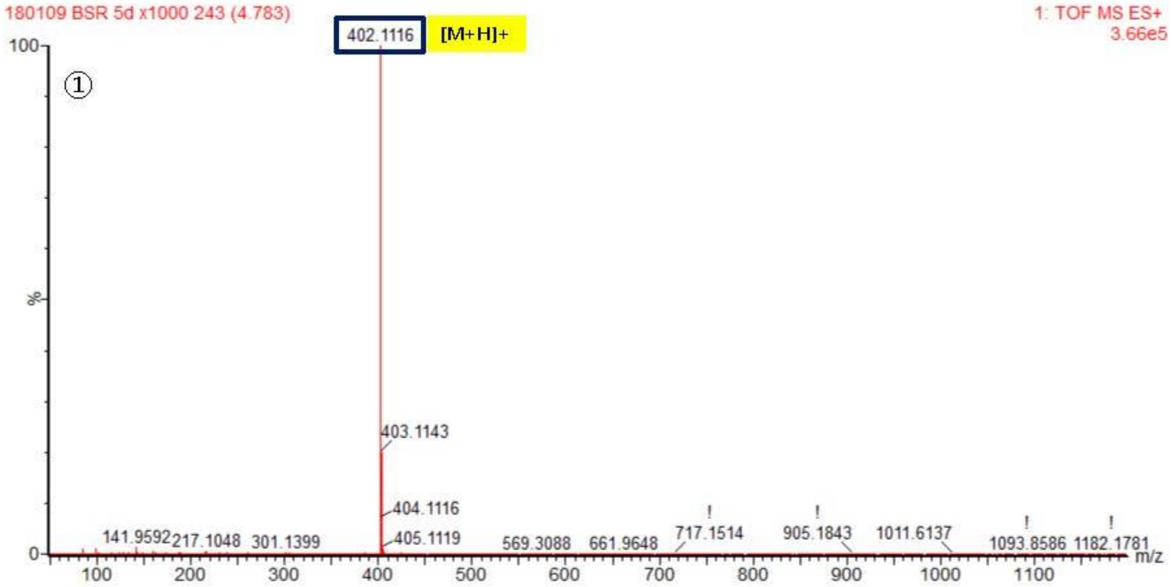

1 NMR spectrum of compound **5c**

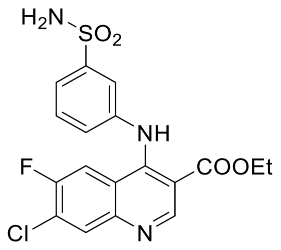

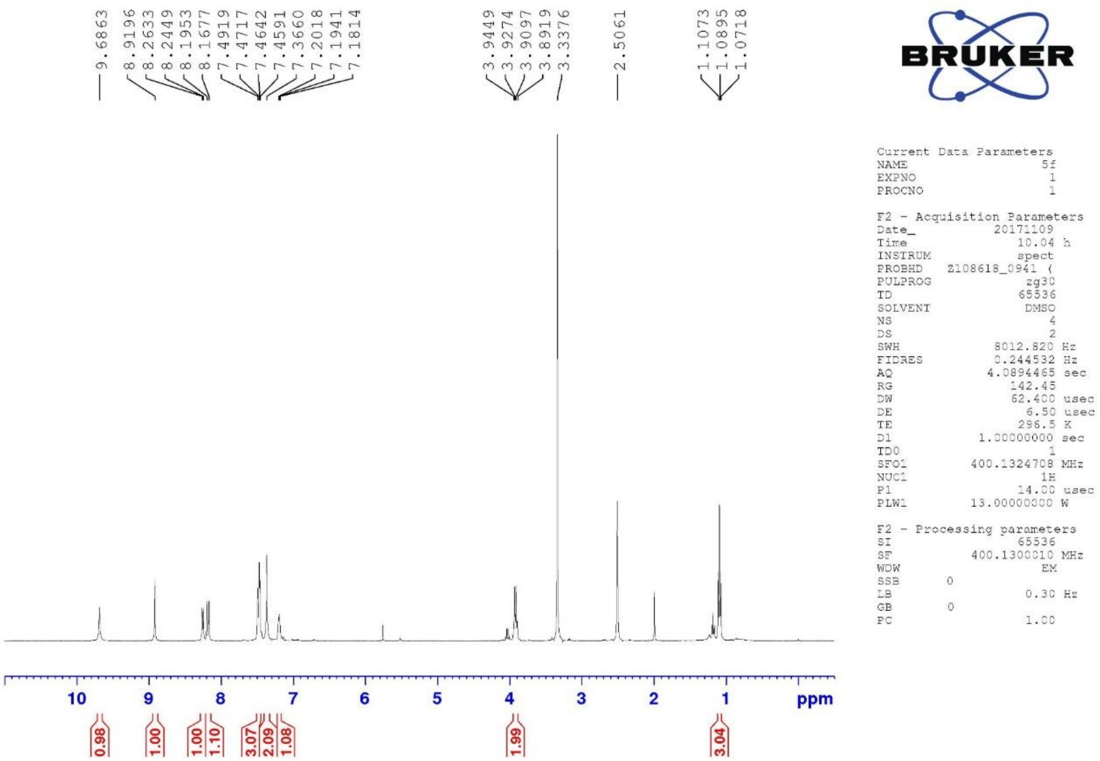

^{13}C NMR spectrum of compound **5c**

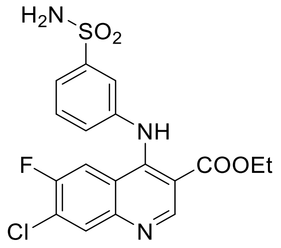

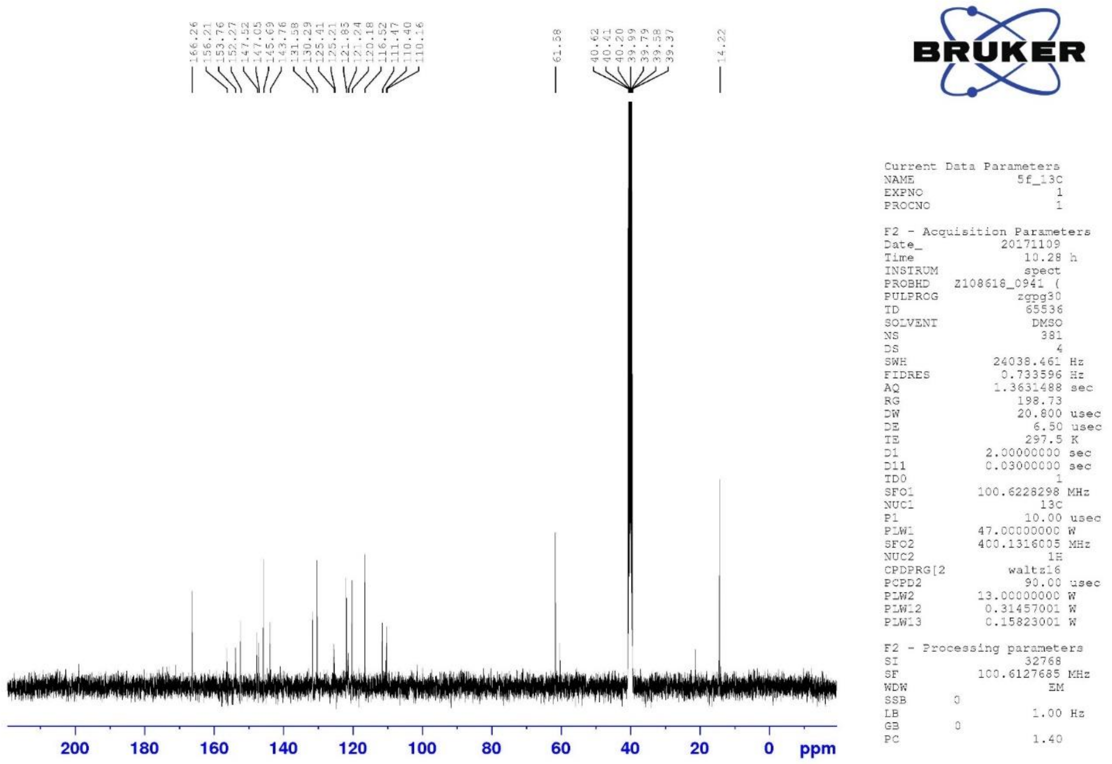

Purity and HRMS of compound **5c**

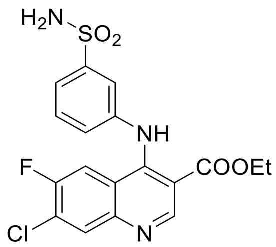

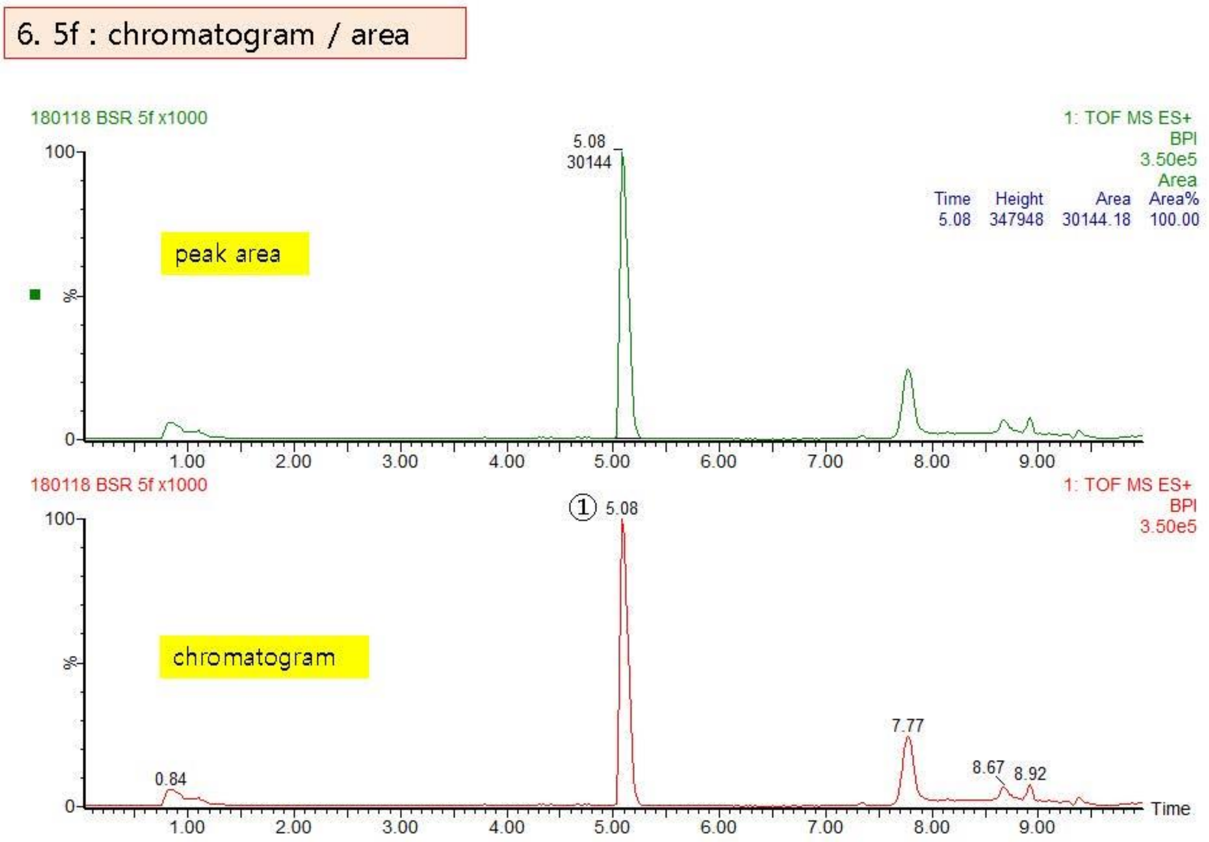

6. 5f : mass spectrum

Molecular formula: $C_{18}H_{15}ClFN_3O_4S$
Exact mass: 423.0456
106.7692 (4+)
142.0230 (3+)
212.5306 (2+)
424.0534 (1+)

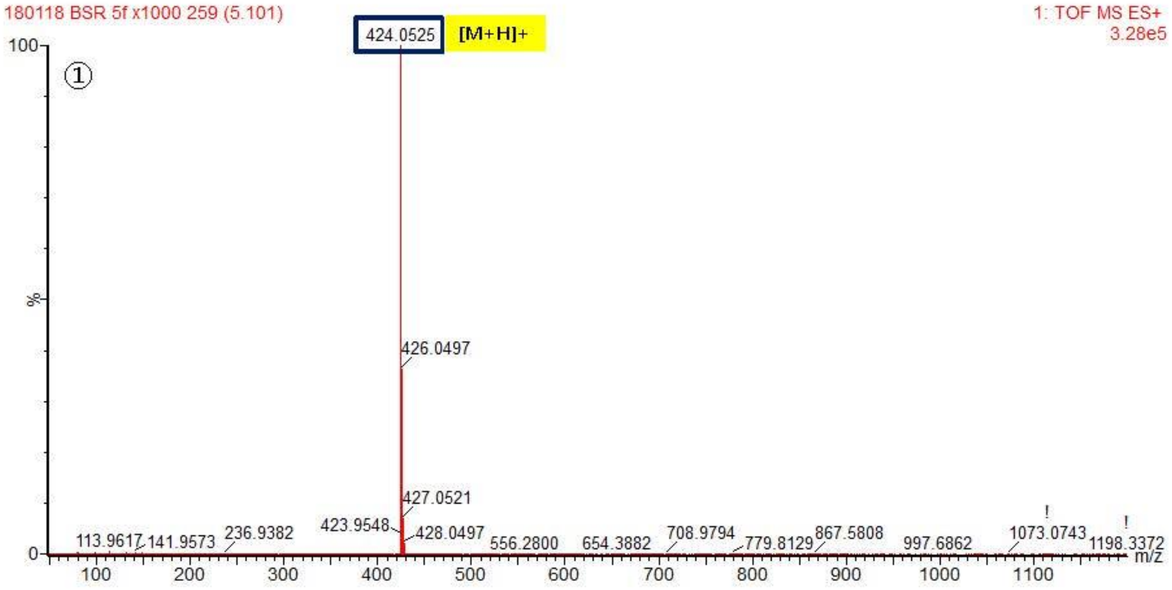

^{1}H NMR spectrum of compound **5d**

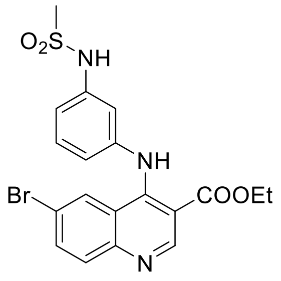

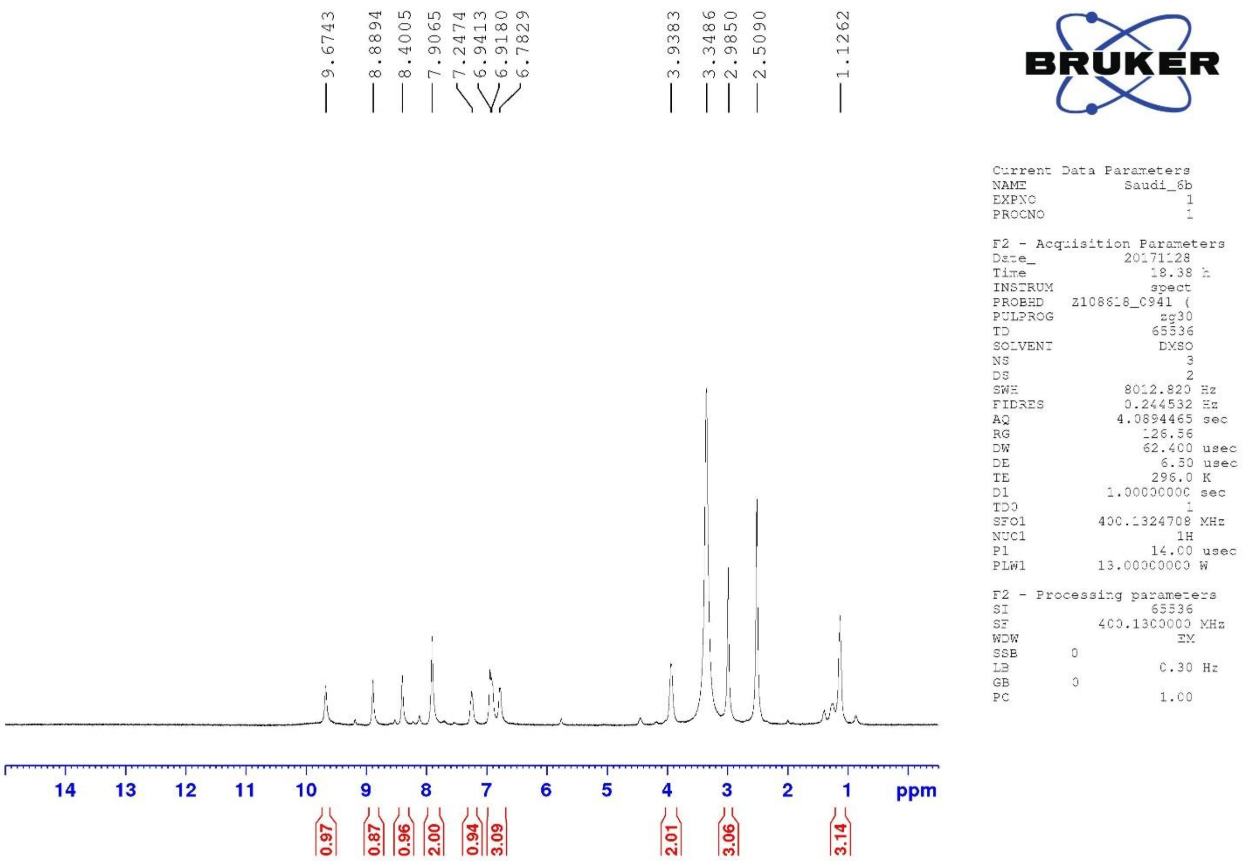

^{13}C NMR spectrum of compound **5d**

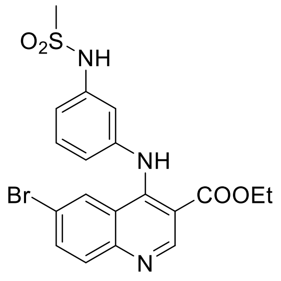

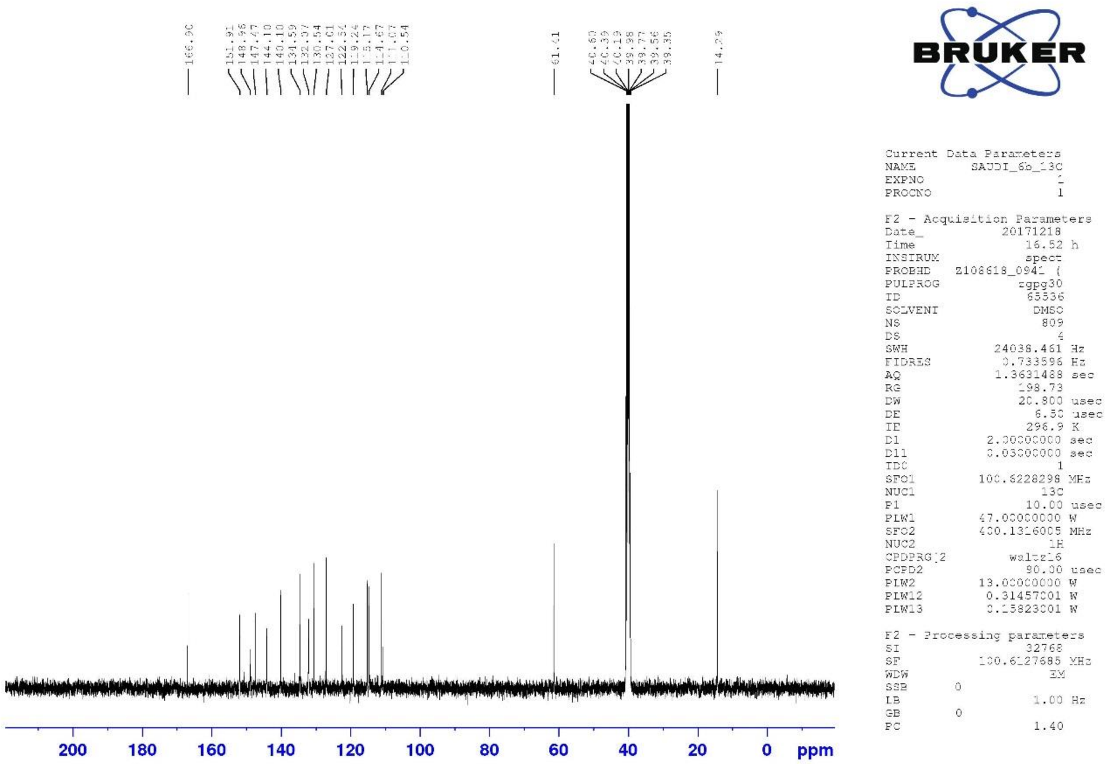

Purity and HRMS of compound **5d**

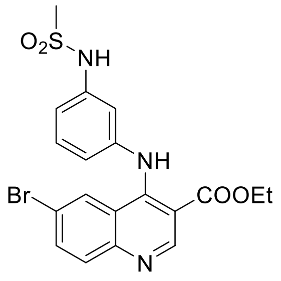

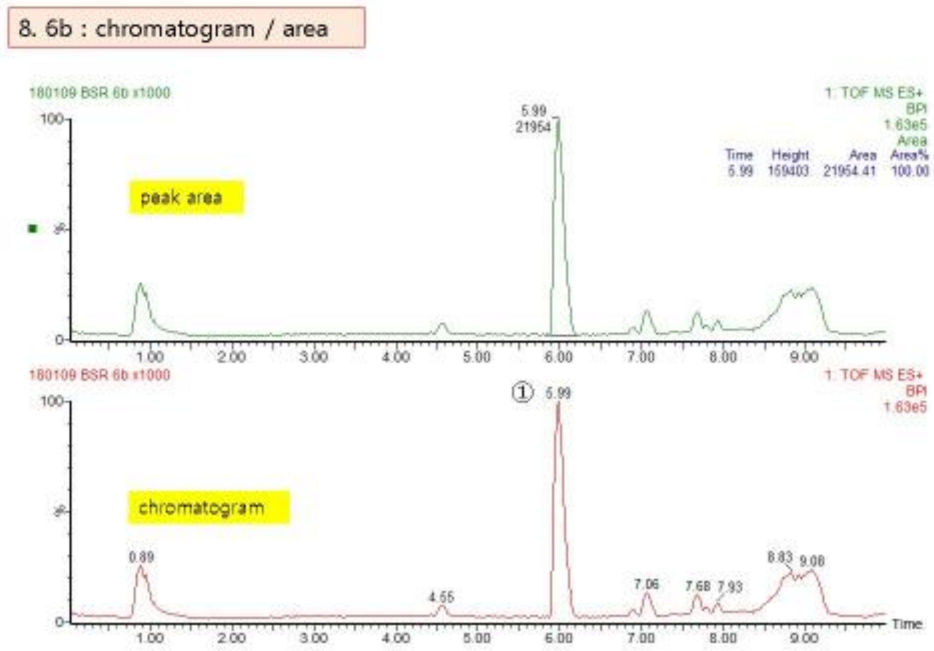

8. 6b : mass spectrum

Molecular formula: $C_{19}H_{18}BrN_{3}O_{4}S$
Exact mass: 463.0201
116.7629 (4+)
155.3479 (3+)
232.5179 (2+)
464.0280 (1+)

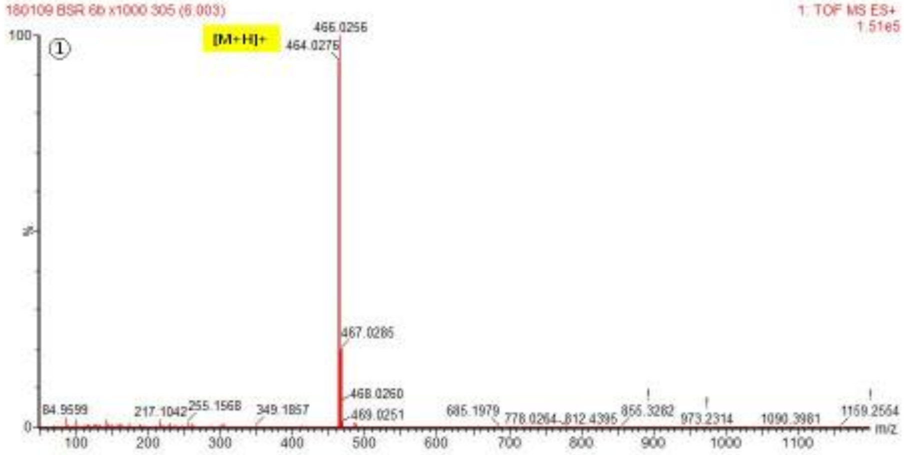

^{1}H NMR spectrum of compound **5e**

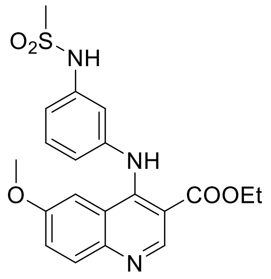

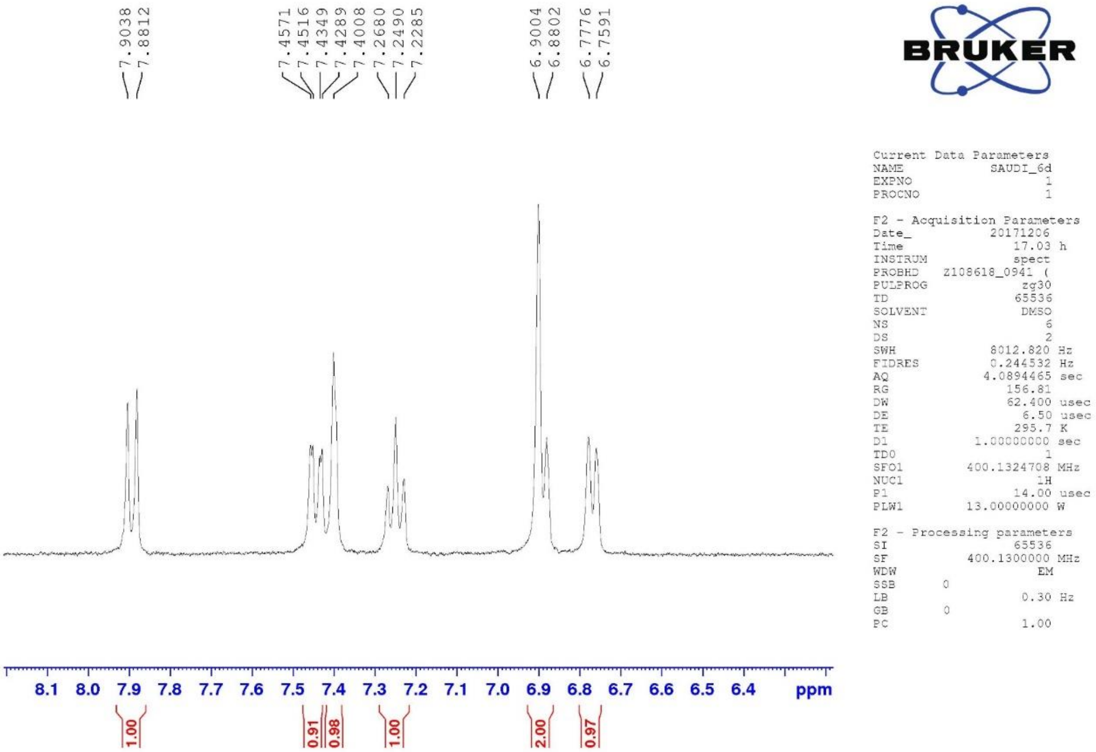

^{13}C NMR spectrum of compound **5e**

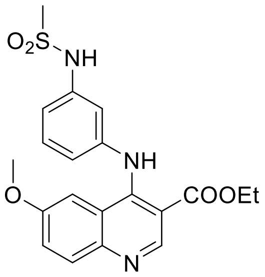

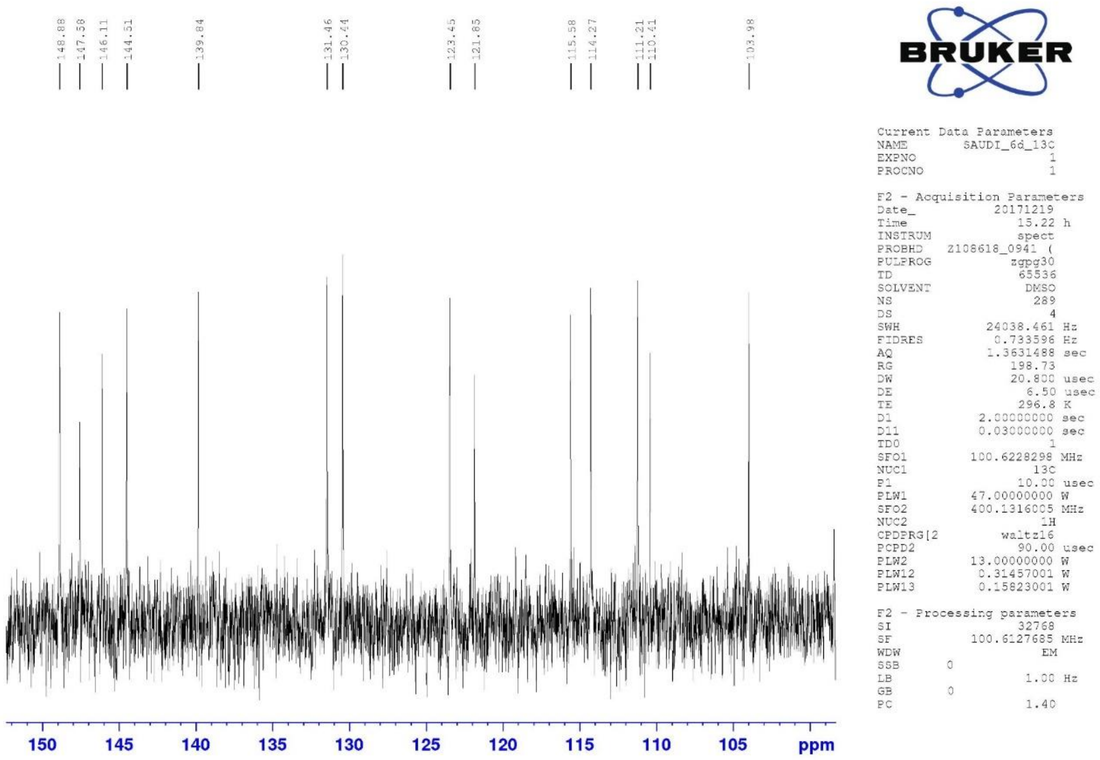

Purity and HRMS of compound **5e**

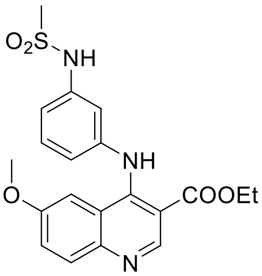

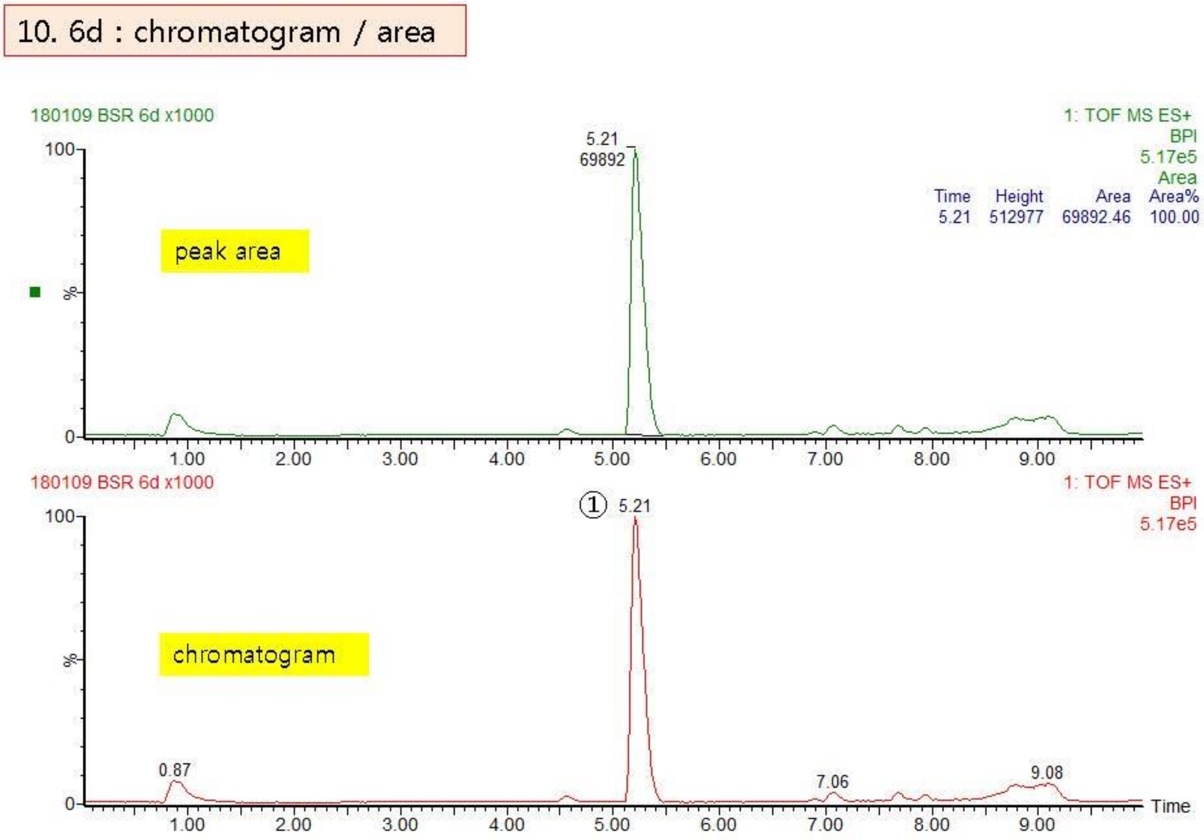

10. 6d : mass spectrum

Molecular formula: $C_{20}H_{21}N_3O_5S$
Exact mass: 415.1202
104.7879 (4+)
139.3812 (3+)
208.5679 (2+)
416.1280 (1+)

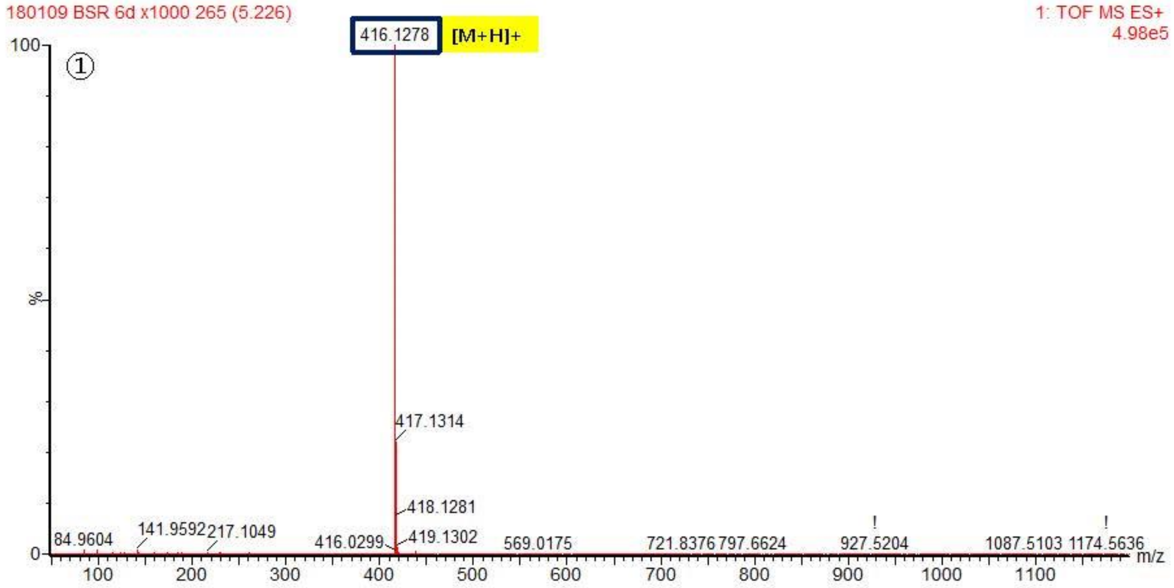

^{1}H NMR spectrum of compound **5f**

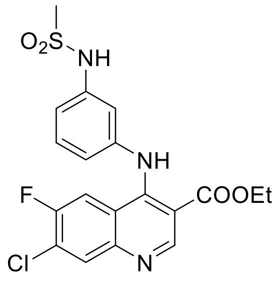

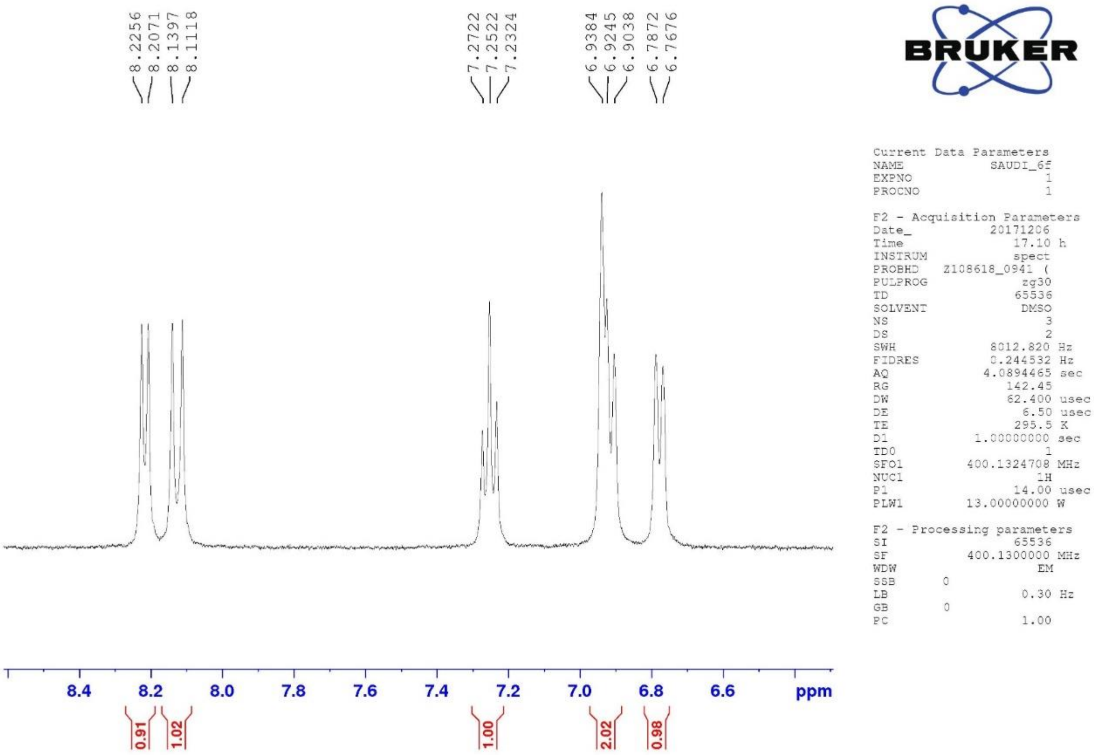

^{13}C NMR spectrum of compound **5f**

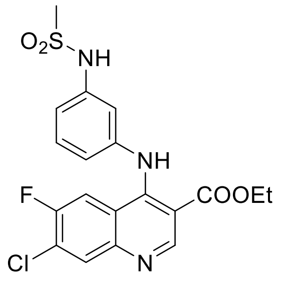

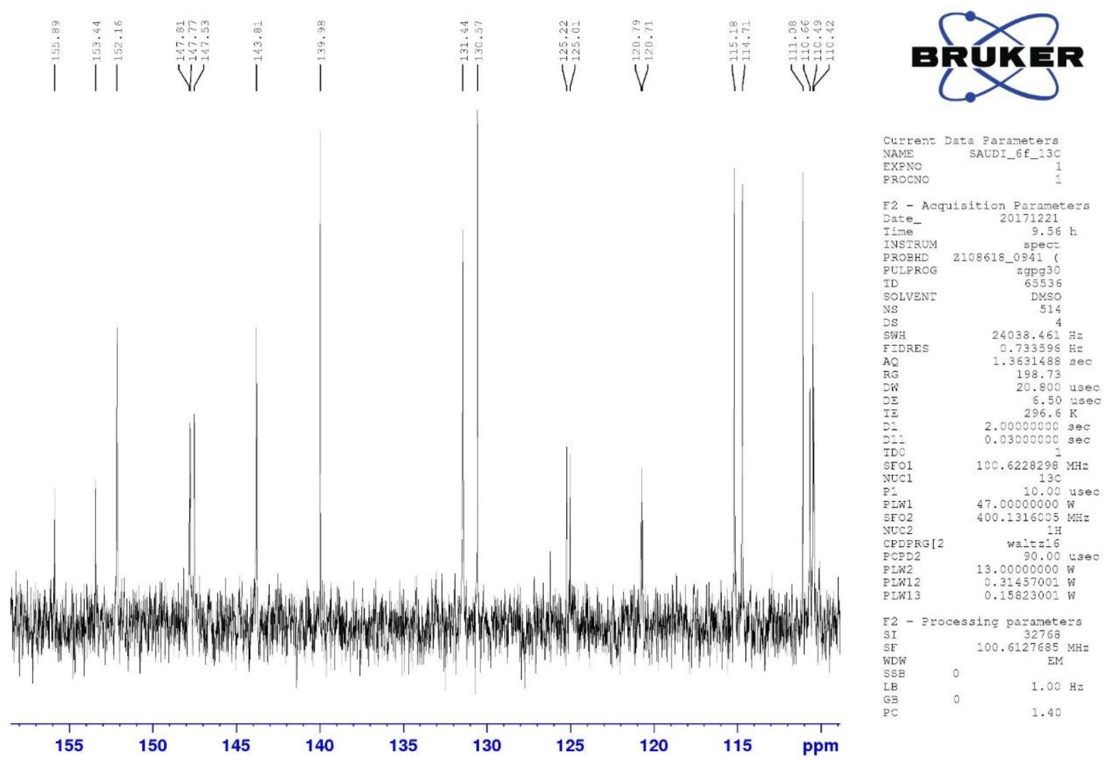

Purity and HRMS of compound **5f**

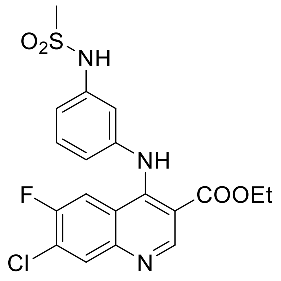

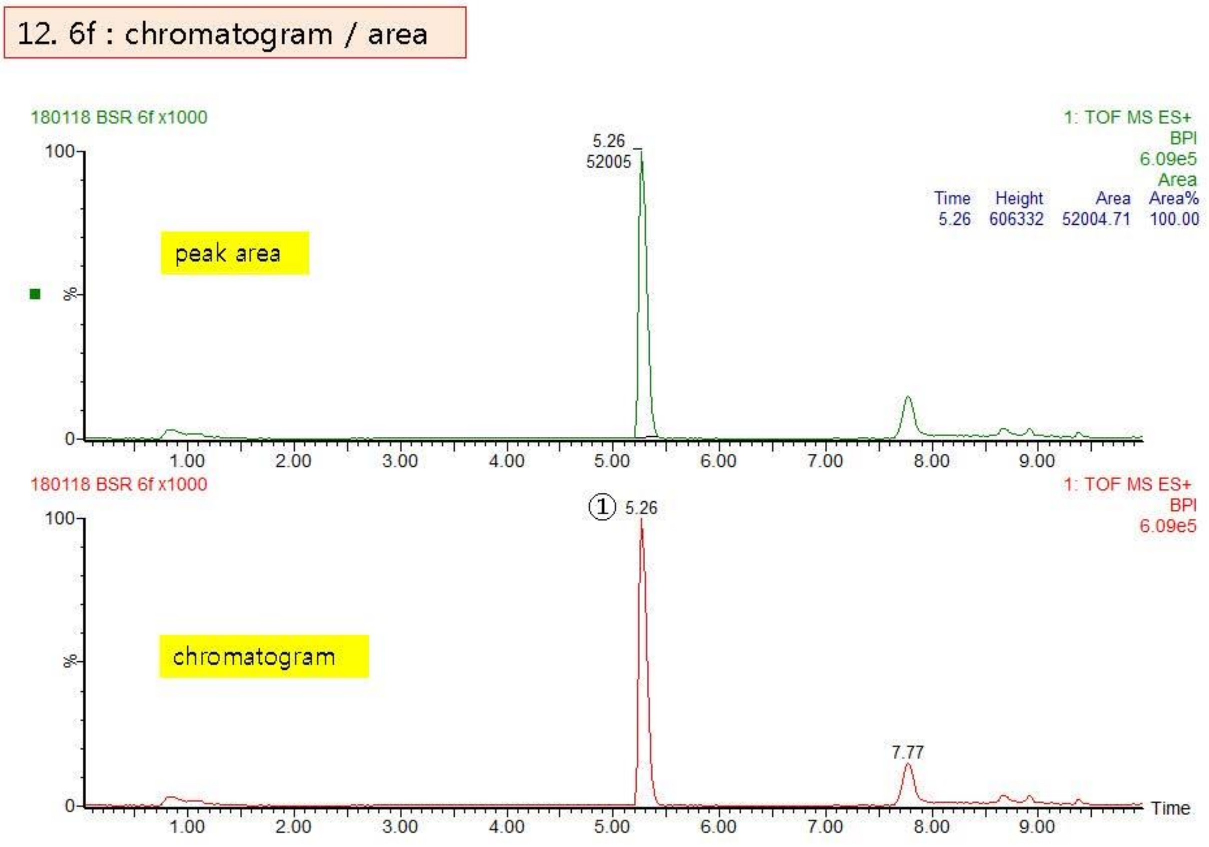

12. 6f : mass spectrum

Molecular formula: $C_{19}H_{17}ClFN_3O_4S$
Exact mass: 437.0612
110.2731 (4+)
146.6949 (3+)
219.5384 (2+)
438.0691 (1+)

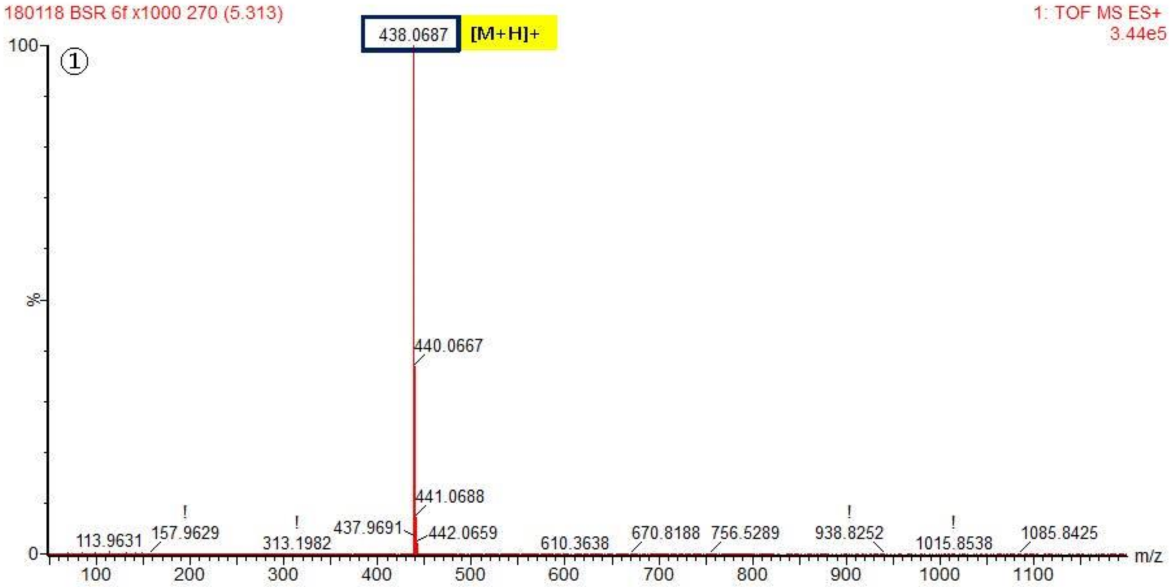

^{1}H NMR spectrum of compound **5g**

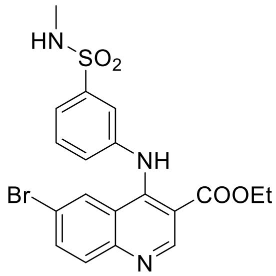

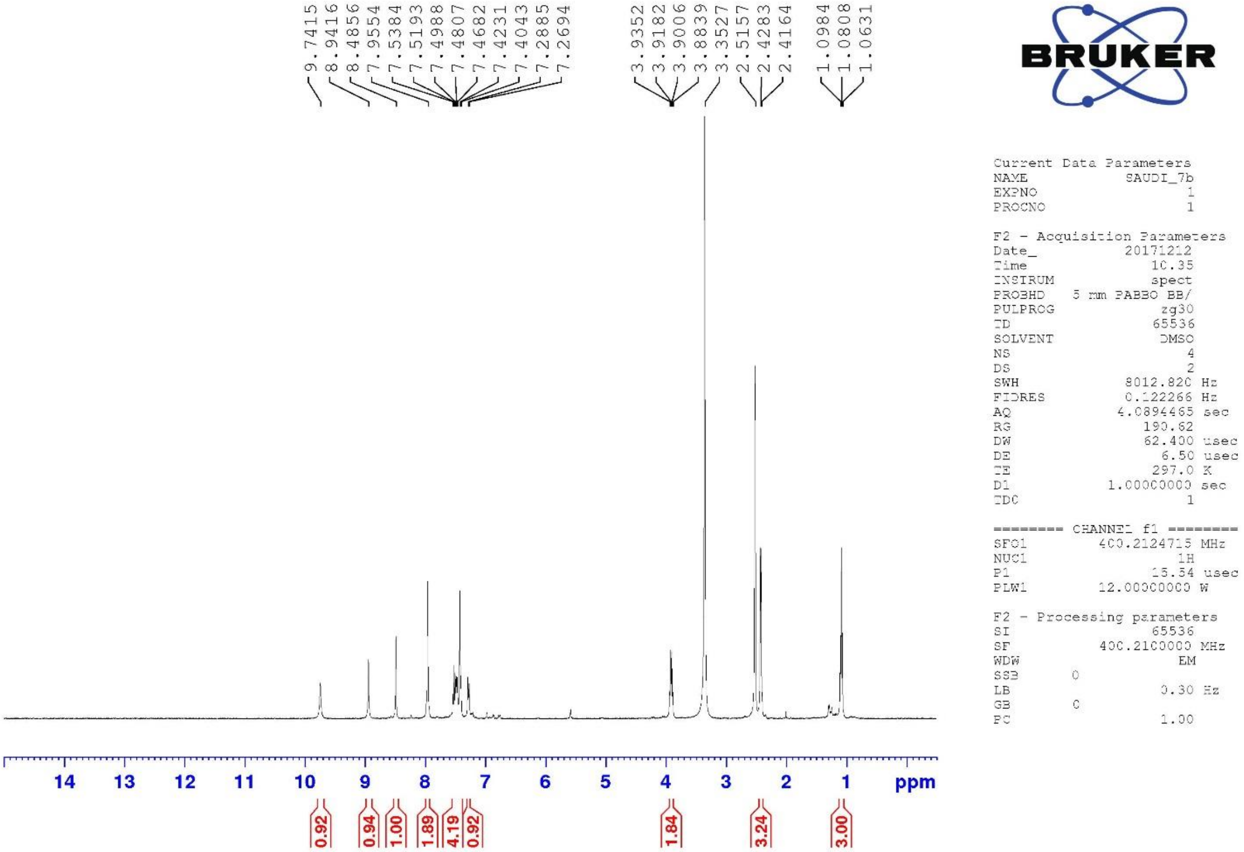

^{13}C NMR spectrum of compound **5g**

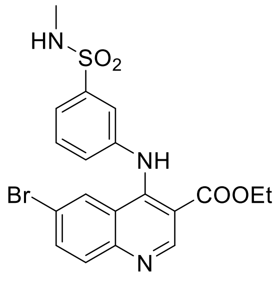

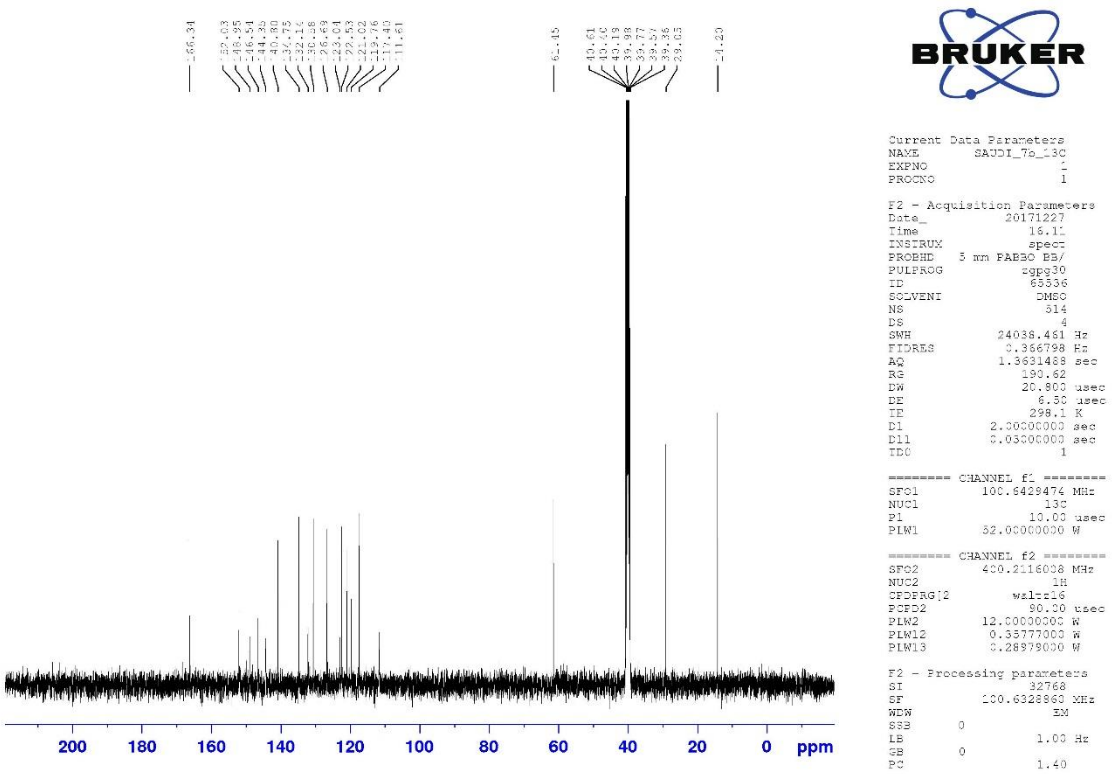

Purity and HRMS of compound **5g**

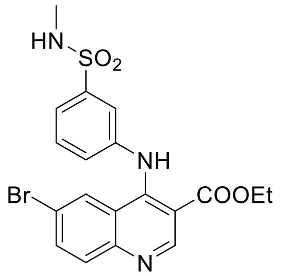

14. 7b : chromatogram / area

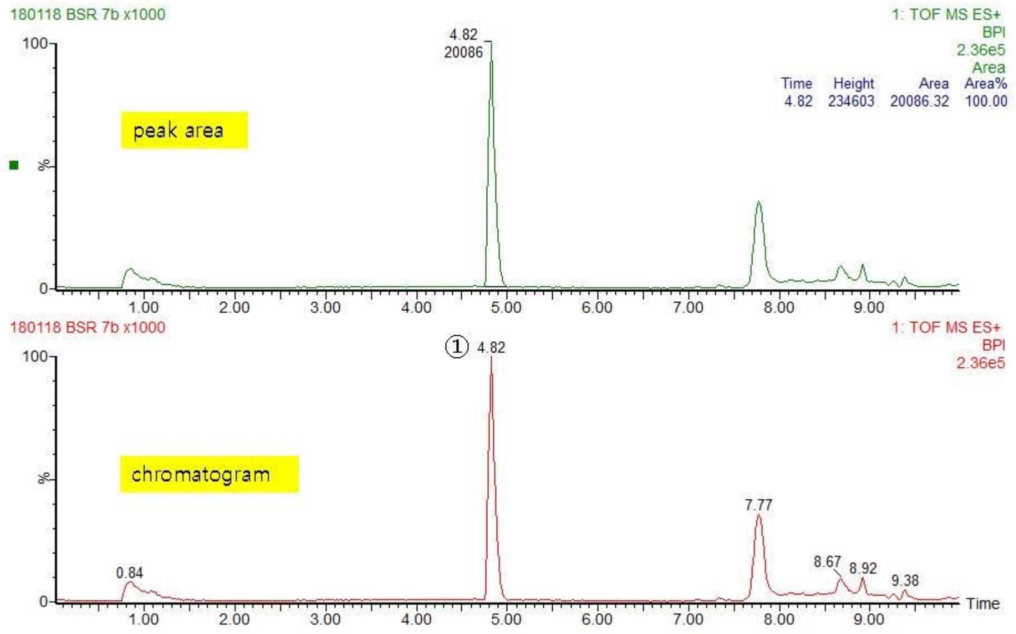

14. 7b : mass spectrum

Molecular formula: $C_{19}H_{18}BrN_3O_4S$
Exact mass: 463.0201
116.7629 (4+)
155.3479 (3+)
232.5179 (2+)
464.0280 (1+)

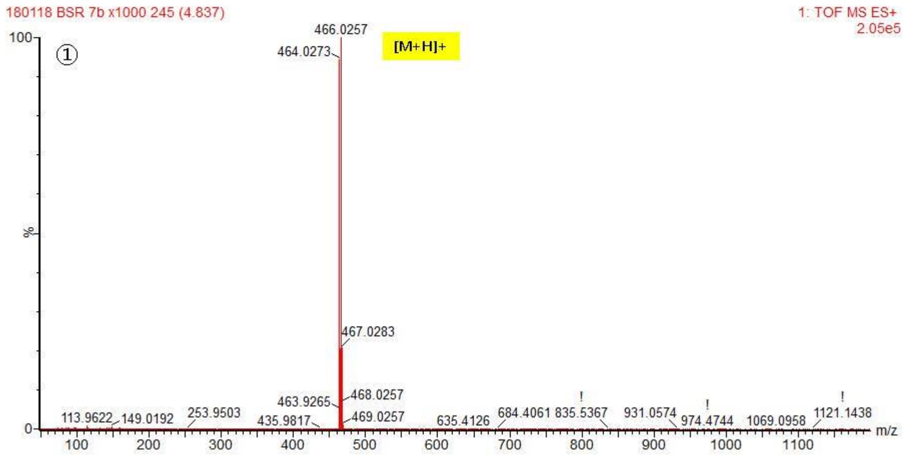

^{1}H NMR spectrum of compound **5h**

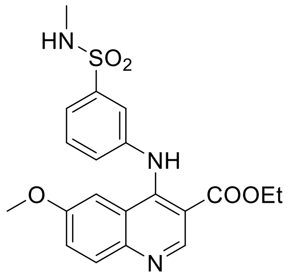

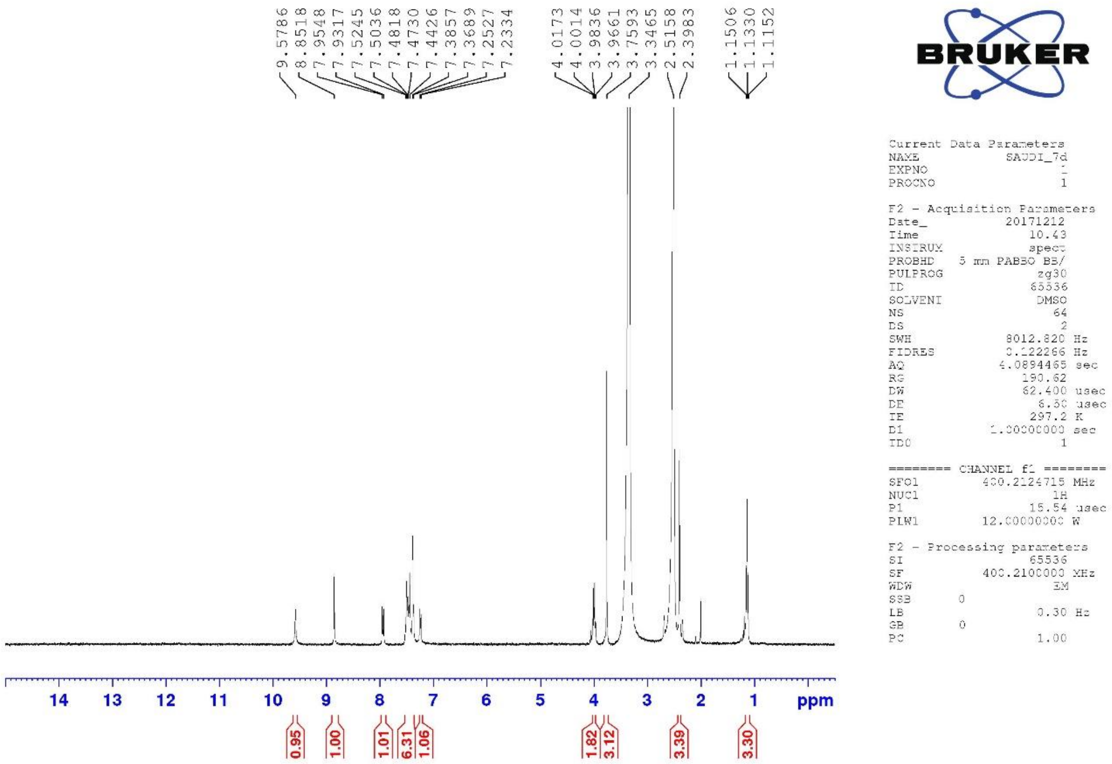

^{13}C NMR spectrum of compound **5h**

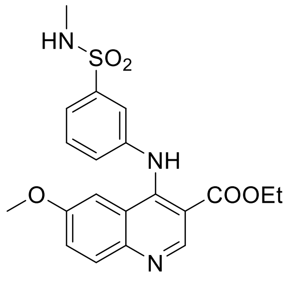

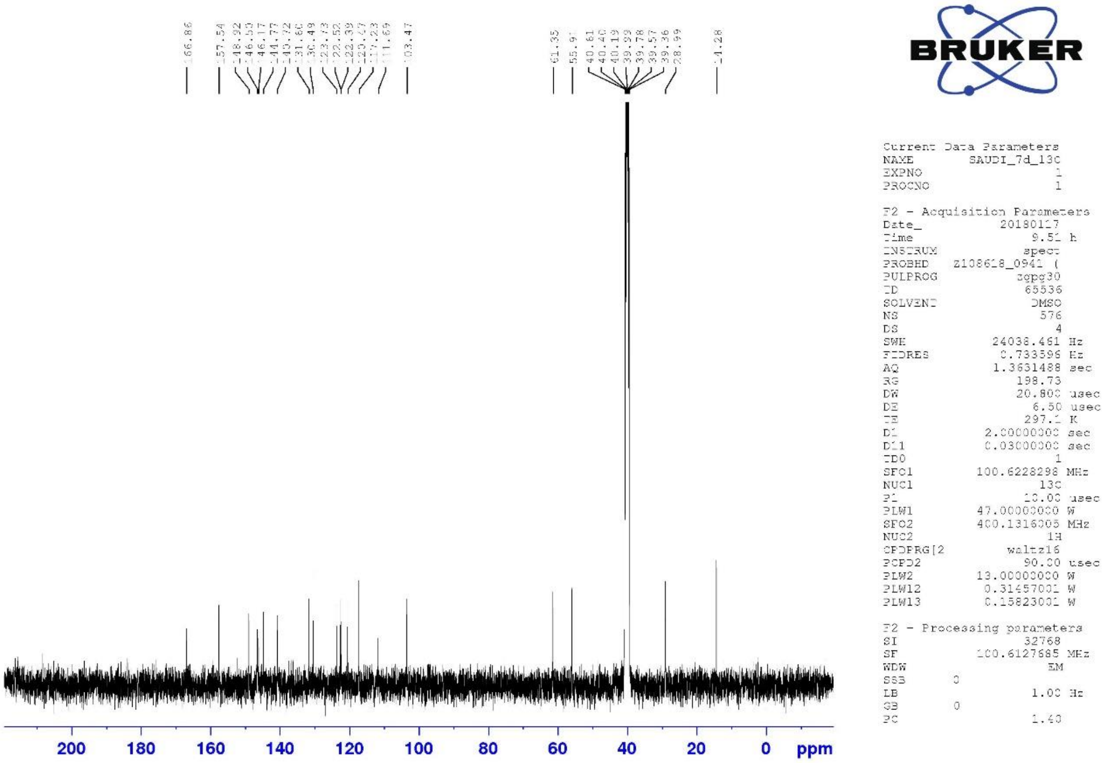

Purity and HRMS of compound **5h**

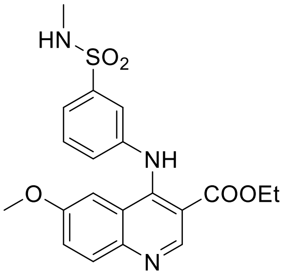

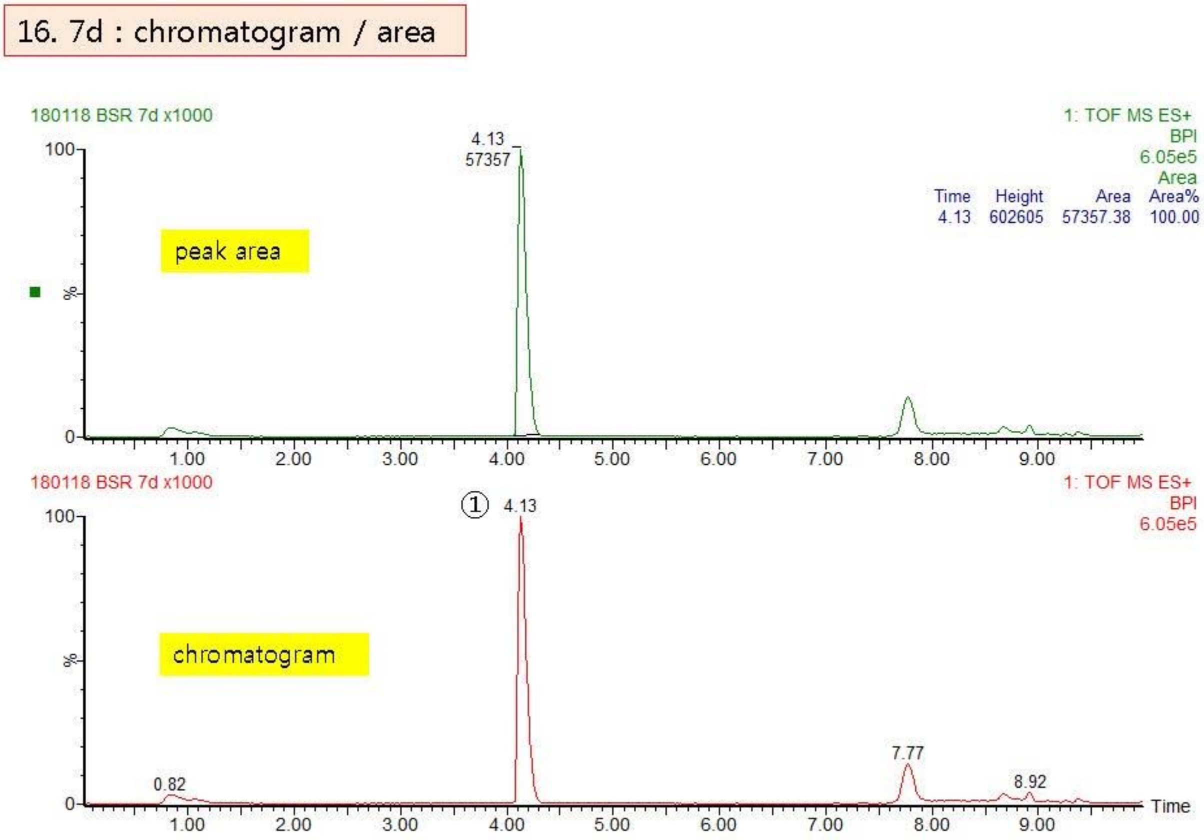

16. 7d : mass spectrum

Molecular formula: $C_{20}H_{21}N_3O_5S$
Exact mass: 415.1202
104.7879 (4+)
139.3812 (3+)
208.5679 (2+)
416.1280 (1+)

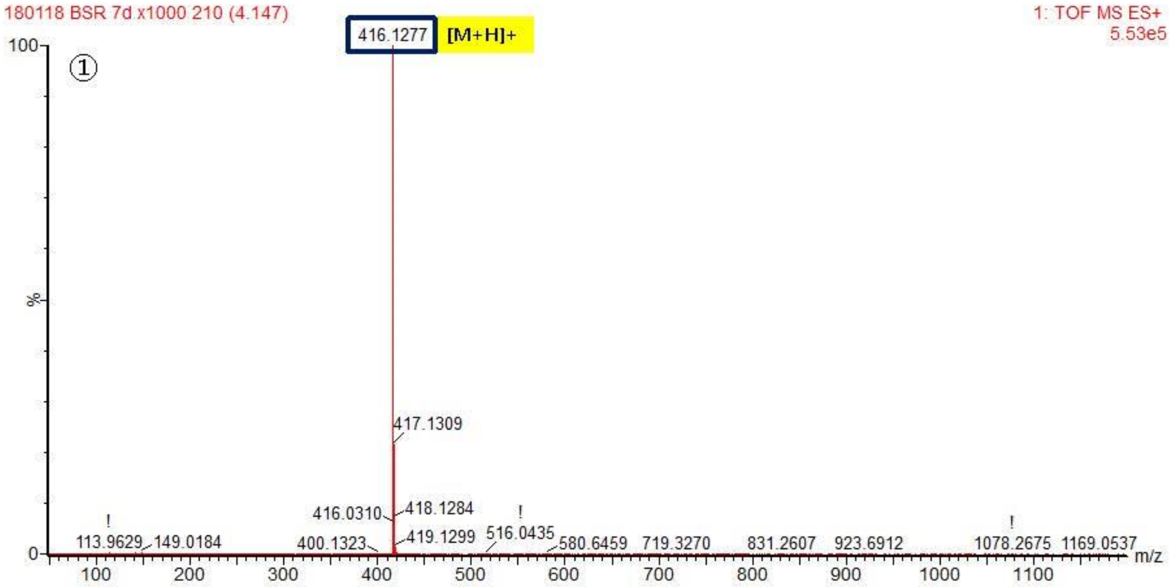

^{1}H NMR spectrum of compound **5i**

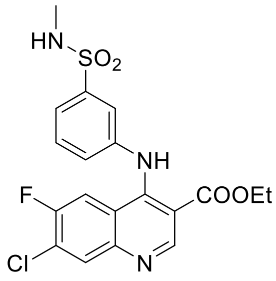

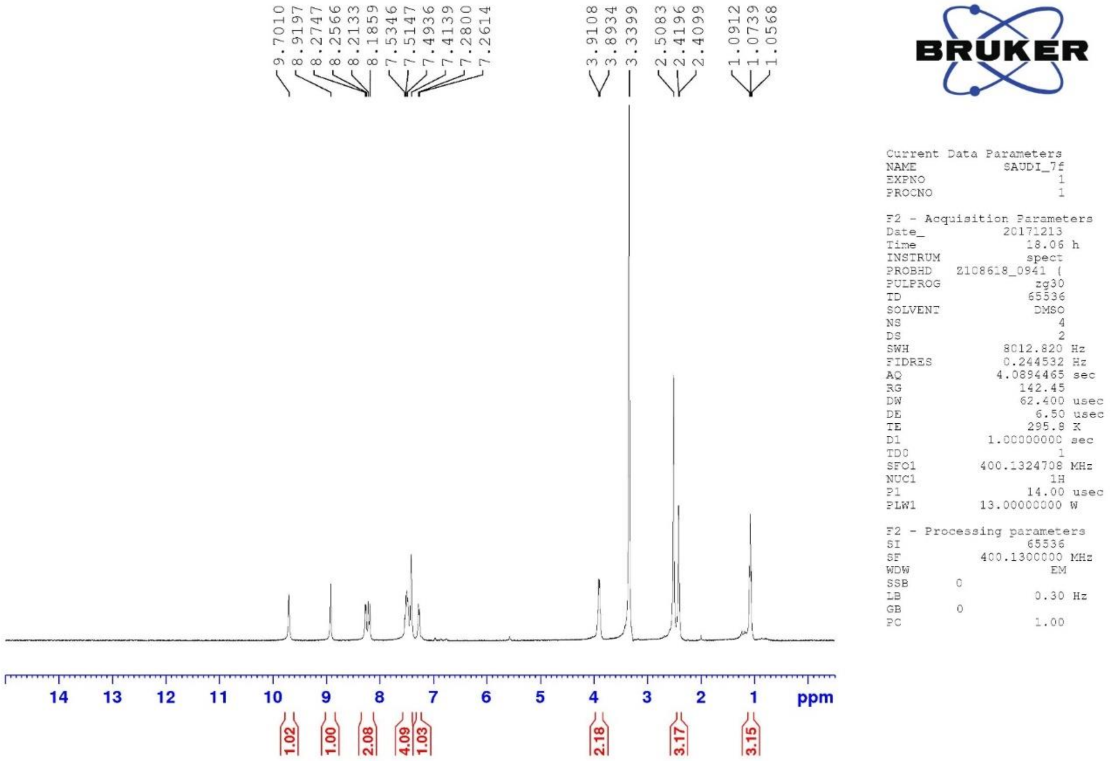

^{13}C NMR spectrum of compound **5i**

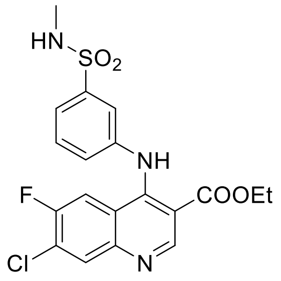

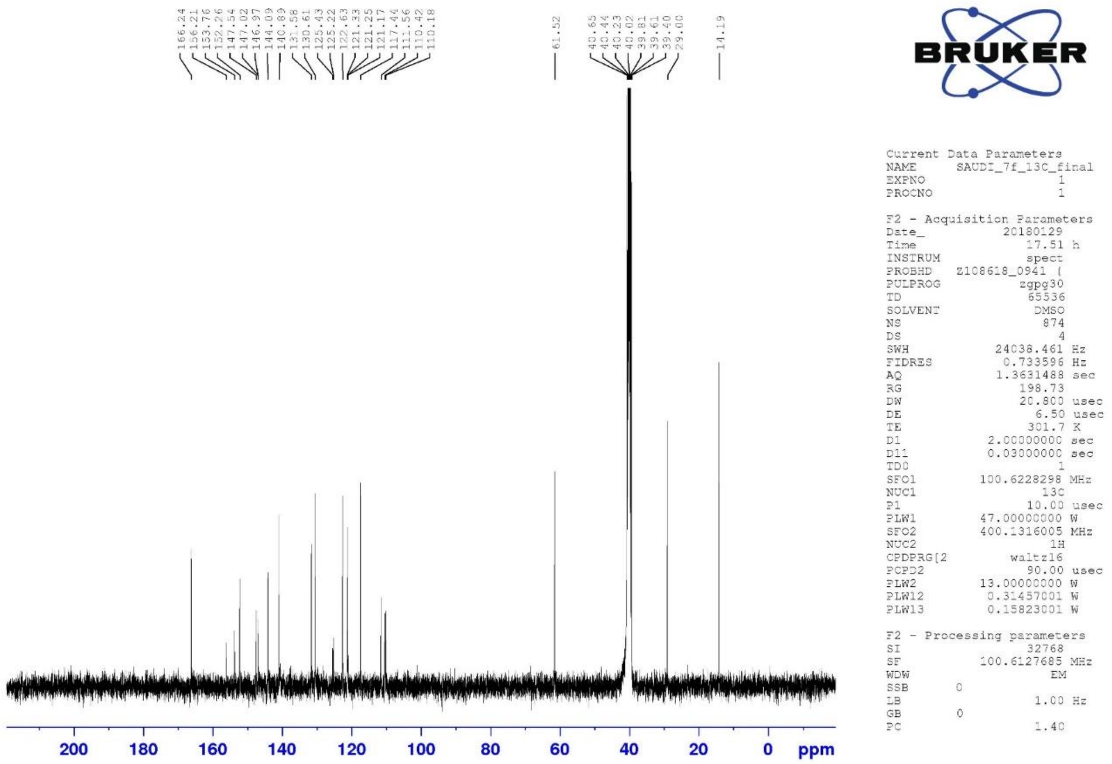

Purity and HRMS of compound **5i**

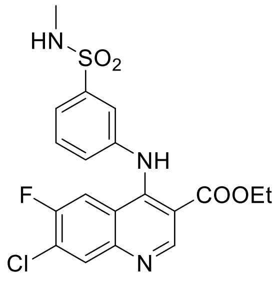

18. 7f : chromatogram / area

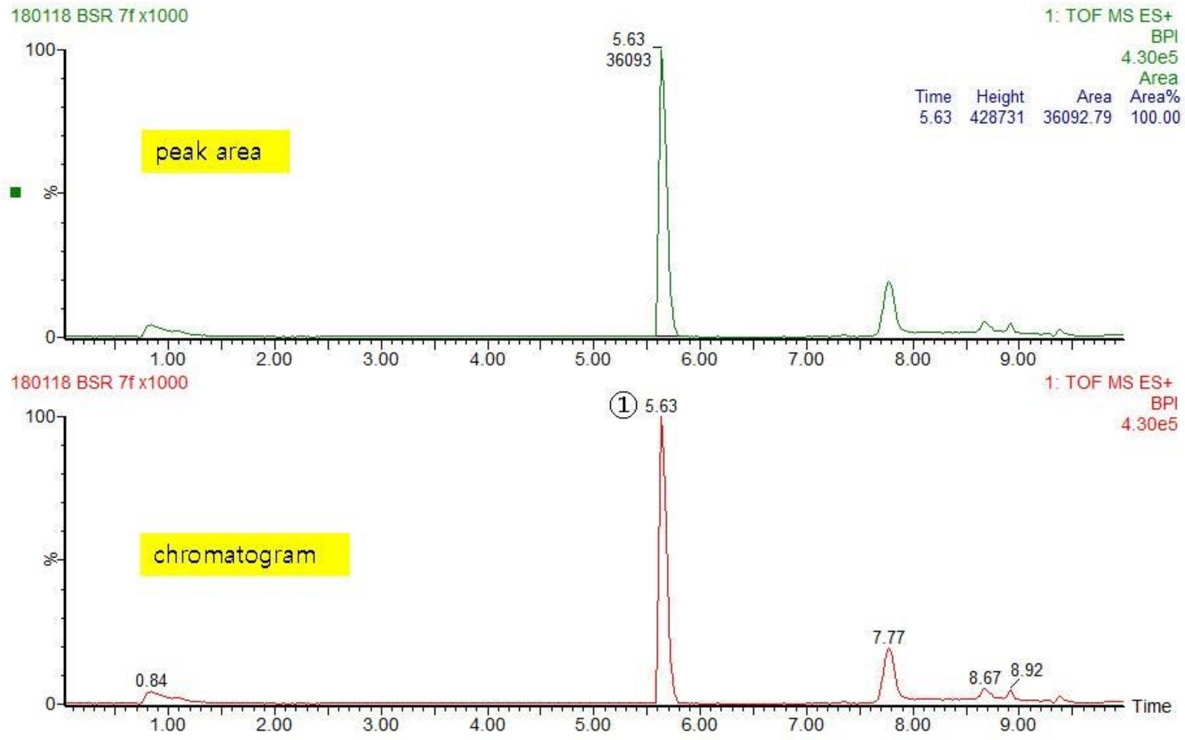

18. 7f : mass spectrum

Molecular formula: $C_{19}H_{17}ClFN_3O_4S$
Exact mass: 437.0612
110.2731 (4+)
146.6949 (3+)
219.5384 (2+)
438.0691 (1+)

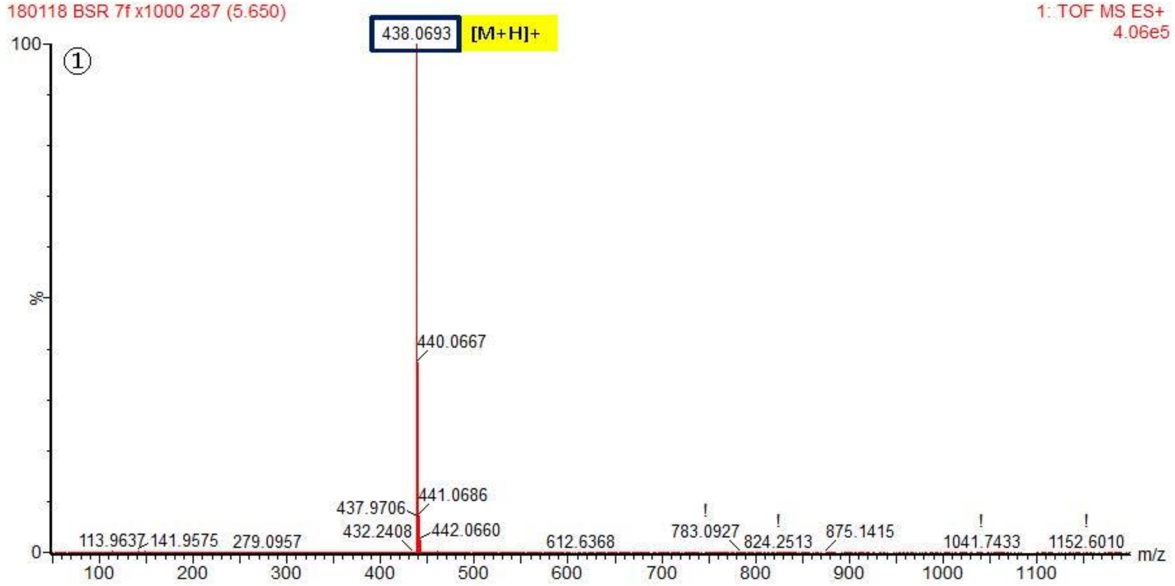

Supplement: Supplemental Information 1 [file peerj-08-8649-s001.pdf]
